# The ROSMAP project: aging and neurodegenerative diseases through omic sciences

**DOI:** 10.3389/fninf.2024.1443865

**Published:** 2024-09-16

**Authors:** Alejandra P. Pérez-González, Aidee Lashmi García-Kroepfly, Keila Adonai Pérez-Fuentes, Roberto Isaac García-Reyes, Fryda Fernanda Solis-Roldan, Jennifer Alejandra Alba-González, Enrique Hernández-Lemus, Guillermo de Anda-Jáuregui

**Affiliations:** ^1^División de Genómica Computacional, Instituto Nacional de Medicina Genómica, Mexico City, Mexico; ^2^Programa de Doctorado en Ciencias Biomedicas, Unidad de Posgrado Edificio B Primer Piso, Ciudad Universitaria, Mexico City, Mexico; ^3^Facultad de Estudios Superiores Iztacala UNAM, Mexico City, Mexico; ^4^Centro de Ciencias de la Complejidad, Universidad Nacional Autónoma de México, Mexico City, Mexico; ^5^Programa de Investigadoras e Investigadores por México Consejo Nacional de Humanidades, Ciencias y Tecnologías (CONAHCYT), Mexico City, Mexico

**Keywords:** omics, Religious Order Study Memory and Aging Project, Alzheimer's disease, aging, genomics, transcriptomics, proteomics, metabolomics

## Abstract

The Religious Order Study and Memory and Aging Project (ROSMAP) is an initiative that integrates two longitudinal cohort studies, which have been collecting clinicopathological and molecular data since the early 1990s. This extensive dataset includes a wide array of omic data, revealing the complex interactions between molecular levels in neurodegenerative diseases (ND) and aging. Neurodegenerative diseases (ND) are frequently associated with morbidity and cognitive decline in older adults. Omics research, in conjunction with clinical variables, is crucial for advancing our understanding of the diagnosis and treatment of neurodegenerative diseases. This summary reviews the extensive omics research—encompassing genomics, transcriptomics, proteomics, metabolomics, epigenomics, and multiomics—conducted through the ROSMAP study. It highlights the significant advancements in understanding the mechanisms underlying neurodegenerative diseases, with a particular focus on Alzheimer's disease.

## 1 Introduction

Neurodegenerative diseases (ND) are becoming an increasingly significant cause of mortality and morbidity, particularly among the elderly. These disorders differ in their epidemiology, clinical manifestations, neuropathology, and treatment approaches. Although individual NDs exhibit diverse clinical presentations and underlying physiological mechanisms, they often share overlapping characteristics (Erkkinen et al., 2018). Diagnostic tests for these diseases are typically expensive, complex, and time-consuming to conduct.

The advent of advanced molecular analysis technologies has revolutionized biological research by enabling the simultaneous investigation of a vast number of biomolecules. Comprehensive profiling of their intricate interactions has paved the way for the emergence of the omics sciences, a flourishing field dedicated to the holistic examination of diverse biological components within organisms (Veenstra, 2021). There has been a substantial increase in the depth and breadth of multi-omics data generated to study Alzheimer's disease (AD). Some examples of projects that assembled together different omic experimental sources are the Alzheimer's Disease Neuroimaging Initiative (ADNI) (Mueller et al., 2005), The Mount Sinai Brain Bank study (MSBB) (Coleman et al., 2023), The Mayo Clinic Brain Bank (MCBB) (Allen et al., 2016), The National Institute on Aging Genetics of Alzheimer's Disease Data Storage Site (NIAGADS) (Kuzma et al., 2016), The National Alzheimer's Coordinating Center (NACC) (Beekly et al., 2004), and the Religious Orders Study and Memory and Aging Project (ROSMAP) (De Jager et al., 2018). Rather than limiting their perspective to a single data type, these collections weave together a multilevel molecular landscape.

This is of relevance since the availability, study and integration of clinical and omics data is essential for biomedical research and the development of precision medicine, especially in the study, prevention and treatment of complex diseases (Jaumot et al., 2018), such as those of neurodegenerative nature. The integration of omics data, facilitated by advanced computational tools, has emerged as a powerful approach for dissecting complex biological systems. This synergistic strategy enables researchers to bridge the gap between molecular pathways and organism function by comprehensively analyzing information across multiple biological layers (Abdelnour et al., 2022; De Jager et al., 2018; Levine et al., 2018). This has the potential to revolutionize diagnostics by facilitating the identification of molecular signatures, novel biomarkers and improving the molecular characterization of diverse pathologies. The use of omics data analysis in studying the nervous system shows promise in understanding the causes of age-related cognitive decline and dementia. This understanding can help develop effective public health strategies and specialized medical care for these common conditions (Livingston et al., 2020).

The Religious Orders Study and the Memory and Aging Project (usually referred to by its acronym ROSMAP) is a longitudinal study composed by two parallel cohort studies that emerged in the early 1990s as a resource for understanding mechanisms underlying aging, memory, cognitive decline, chronic diseases of aging, and other health outcomes. Both studies are specifically designed to study aging and risk factors for cognitive decline and incident Alzheimer's type dementia. Since 1994, it has recruited individuals over the age of 65 in two groups: the ROS (Religious Order Study) group, which studies nuns, priests and lay people from across the United States, and the MAP (Memory and Aging Project) group, which studies lay people from across northeastern Illinois. Both cohorts are standardized for clinical, mental, genetic, imaging and other evaluations on an annual basis and there is an organ donation agreement at the time of death for access to the study. Donation includes brain, spinal cord, nerve and muscle in the case of autopsies (Bennett D. A. et al., 2018). The value of this longitudinal study design is that it allows us to see the progression and changes over time in the molecular, physiological and clinical characteristics of patients, opening a panorama that allows the study of diseases of aging from pathogenesis to death of individuals, since it is known that diseases of cognitive impairment can present pathophysiological changes in the brain many years before the clinical manifestations of the disease (Beason-Held et al., 2013; Reiman et al., 2004) and appear on a spectrum ranging from clinically asymptomatic to severely impaired (Tahami Monfared et al., 2022).

In this regard, cataloging multi-omics data on ROSMAP subjects, regardless of their health status trajectory, may provide insights into molecular events that contribute to aging-related cognitive impairment (Bennett D. A. et al., 2018). The ROSMAP discovery pipeline takes advantage of the availability of a multilevel omics dataset generated from postmortem frozen brain tissue from the dorsolateral prefrontal cortex (DLPFC) of non-Hispanic white participants. The choice of the DLPFC region as the focus of omics analysis stems from the overriding consideration of identifying a region implicated in a multiplicity of pathological processes and conditions related to mechanisms of cognitive impairment in aging and cognitive pathologies.

Although the focus of this project is on AD, research and applications extend far beyond to include cardiovascular disease (CVD), dementia with Lewy bodies (LBD), Parkinson's disease (PD) and normal pressure hydrocephalus (HS), among others (Bennett et al., 2014).

**Figure 1:**
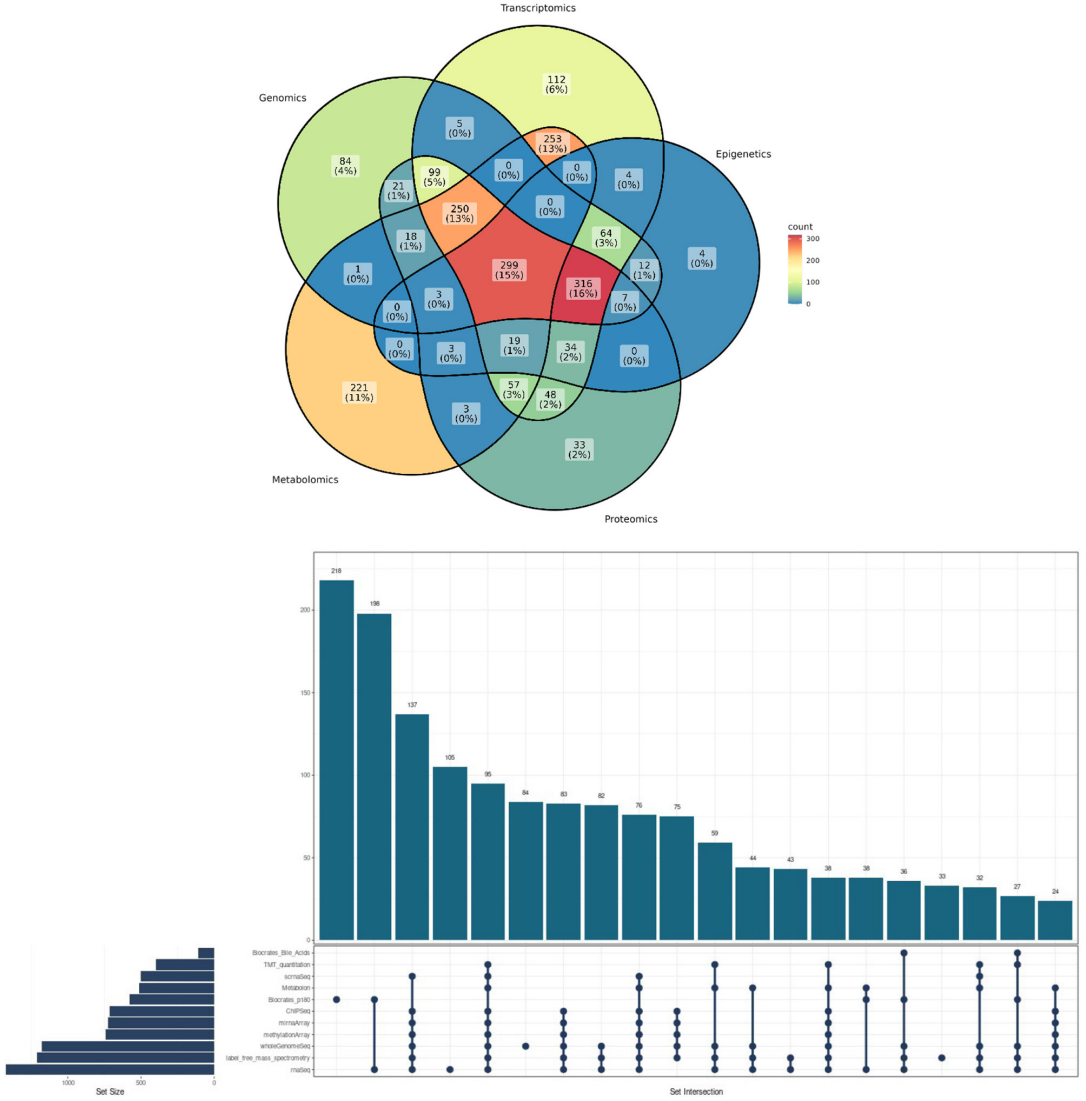
Methodological convergences in the ROSMAP cohort omic data. **(Top)** Venn Diagram showcasing the percentage of convergence in omic analysis made to ROSMAP participants. **(Bottom)** Upsetplot describing the specific technologies used for the ROSMAP database, the number of samples for each technology and the convergence and divergence of participants in one or more technologies.

As of this review's date, a total of 2,557 individuals from both cohorts (70.8% women and 29.2% men) have participated in omics technology trials, of which 10,379 samples have been analyzed, mainly from the DLPFC (see Table 1; Figure 1). Among those studied, 97.49% were of Caucasian origin, 1.9% were African American, 0.11% were Native American and the rest belonged to other racial groups. Of the people studied using omics technologies, 33.39% received no clinical diagnosis of dementia at the time of death, in contrast, about 24.79% were diagnosed with Alzheimer's dementia, with no other form of cognitive impairment.

**Table 1:** Table summarizing demographic data and the availability of omic assays stratified by CERAD score criteria, a neuropsychological battery which provides a measurement of AD progression (Rossetti et al., 2010), in ROS and MAP.

	**Alzheimer's disease (26.62%)**	**Non-Alzheimer's disease (73.37%)**	**Total (*n* = 2,557 individuals with omic assays)**
Sex	50.36 % males, 49.64 % females	49.86% males, 50.14% females	1,810 females, 747 males
APOE4 status	32.16 % APOE4+	77.09 % APOE4+	24.86 % APOE4+
Mean years of education	17.12 for males, 15.78 for females	16.98 for males, 15.77 for females	16.2 years on average

Data was obtained from synapse entry syn2580853 and the AD Knowledge Portal.

**Figure 2:**
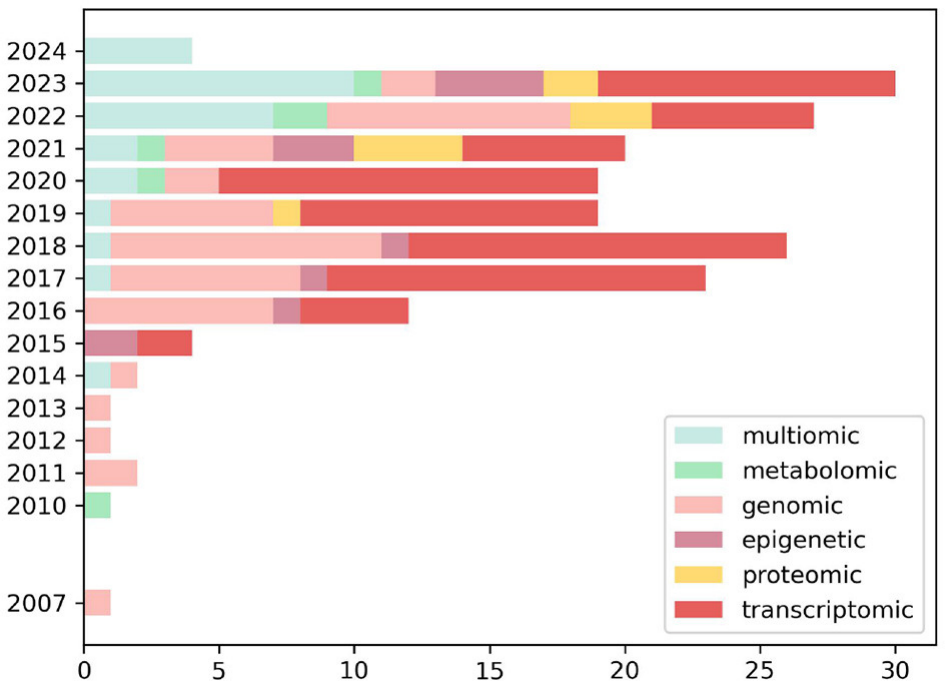
Number of articles published annually from 2007 to 2024, classified by different omics technologies within a specific cohort. The categories of omics technologies include genomics, transcriptomics, epigenetics, proteomics, metabolomics, and multi-omics. A notable growth in the use of multi-omics approaches in recent years is highlighted, reflected by an increase in the number of integrative studies spanning multiple omics disciplines. This increase suggests a trend toward the adoption of more integrated and multidimensional analyses in biomedical research.

ROSMAP has been crucial as a discovery, and as a replication cohort to establish associations related to dementias, exploring the relationships between various biological factors and cognitive decline or AD. These factors encompass genetic variants, gene expression profiles, proteomic signatures, epigenetic modifications, and metabolomic profiles (see Figure 2). Additionally, investigations have delved into regulatory factors, risk factors for cognitive decline, motor function, molecular interactions between these elements, and even the potential for translating such findings into drug development (Bennett D. A. et al., 2018; Bennett et al., 2014).

In this context, different types of omics information have been generated and/or analyzed such as DNA methylation (He et al., 2021; Ng et al., 2017), H3K9ac ChIP-Seq (Klein et al., 2019), RNA-seq (Li et al., 2023; McCorkindale et al., 2022; Patrick et al., 2017; Shulman et al., 2023; Tasaki et al., 2022; Patrick et al., 2017), miRNAs (Jurkiewicz et al., 2020; Lugli et al., 2015; Patrick et al., 2017), nuclear and mitochondrial whole genome sequencing (Klein et al., 2021; Vialle et al., 2022), scRNA-seq (Mathys et al., 2019), single nucleus RNA (snRNA) (Seto et al., 2023), proteomics (Carlyle et al., 2021; Johnson et al., 2020; Wingo et al., 2020; Yu et al., 2019), metabolomics (Mufson and Leurgans, 2010; Varma et al., 2021; Wang et al., 2020), in brain, blood tissue and immune cells. Mentioned data is available in platforms such as http://www.radc.rush.edu, http://www.synapse.org, and http://www.niagads.org, via request.

This review article aims to explore the current and potential impact of the study of omic data derived from ROSMAP. We will focus on analyzing the advances achieved in the fields of genomics, proteomics, transcriptomics, metabolomics and epigenetics of AD and related diseases derived from the study of several laboratories around the world that have used the cohort data in order to understand its influence and impact on the investigation of phenomena associated with the aging process. A brief meta-analysis of the topics studied concerning Alzheimer's disease using ROSMAP data, as well as outside of the cohort, can be found in the Supplementary material.

## 2 Genomics

In diseases of aging, such as Alzheimer's dementia, genes and genetic variants play a substantial role in the development and pathogenesis (Bertram and Tanzi, 2020; Dumitrescu et al., 2020). The value of genomic analyses lies in the fact that they can provide a panoramic view of diverse biological processes beyond regulation. Classically, variants in genes such as APP, PSEN1 and PSEN2 are known to cause autosomal dominant Alzheimer's dementia (Dai et al., 2017). Moreover, several genetic variants have been associated with neuropathological events in late onset AD (LOAD), like APOE (Rebeck et al., 1993) including variants related to pathological processing of Tau and Aβ, neuroinflammation, oxidative stress, and the occurrence of neuronal, synaptic and mitochondrial dysfunction (Andrade-Guerrero et al., 2023; Nasb et al., 2024).

**Figure 3:**
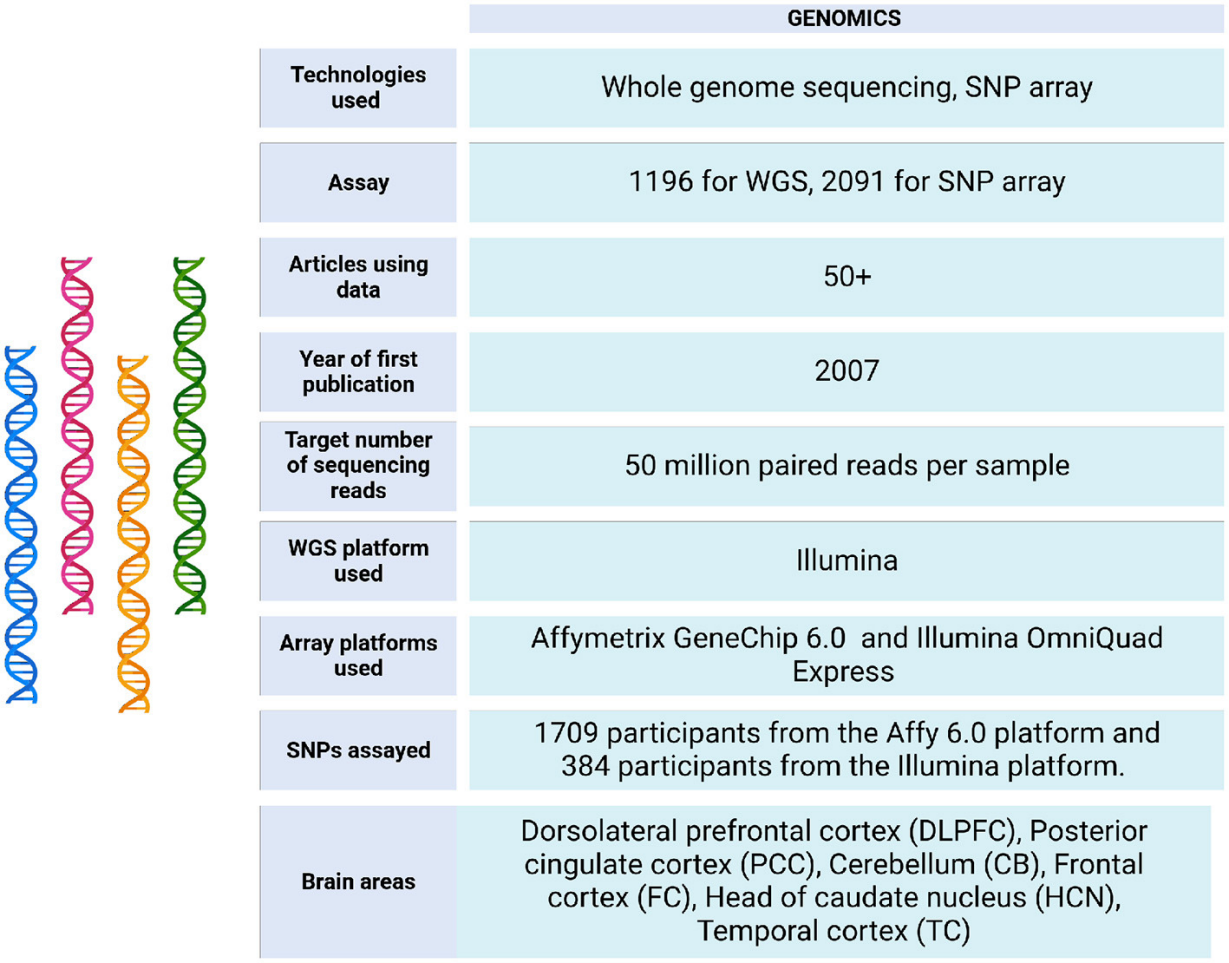
Genomic data metrics in ROSMAP. A subset of the ROSMAP samples (*n* = 1,200, representing 1,179 unique deceased participants) underwent whole genome sequencing (WGS). DNA was extracted from brain tissue (*n* = 806), whole blood (*n* = 389), or EBV-transformed lymphocytes (*n* = 5). The WGS libraries were prepared and sequenced on an Illumina HiSeq X sequencer (v2.5 chemistry) using 2 × 150 bp cycles. Variants were annotated with population frequencies from established variant databases, including dbSNP, 1,000 Genomes, and the Exome Aggregation Consortium (ExAC) (De Jager et al., 2018). Only 1,196 bam and bai files are available in https://www.synapse.org/Synapse:syn20068543. Also, the WGS Harmonization study is available in https://www.synapse.org/Synapse:syn22264775. For genotyping, the majority of samples were genotyped on the Affymetrix GeneChip 6.0 platform at the Broad Institute's Center for Genotyping (*n* = 1,204) or the Translational Genomics Research Institute (*n* = 674). Additionally, 566 participants were genotyped on the Illumina OmniQuad Express platform at Children's Hospital of Philadelphia (De Jager et al., 2018). SNP Array data can be accessed via https://doi.org/10.7303/syn3157325.

The ROSMAP project initially analyzed brain tissues using genotyping and arrays. More recently, it has incorporated next generation sequencing (NGS) technologies, including whole genome sequencing (WGS), to enhance its research capabilities (see Figure 3).

### 2.1 GWAS and gene associations

Primarily through genome-wide association analysis (GWAS), the ROSMAP project has identified genes and genetic variants associated with an increased risk of Alzheimer's disease. For example, rare alleles of the GAB2 gene (Mez et al., 2017; Reiman et al., 2007) or variants in PSEN1, such as p.E318G, have been linked to an increased burden of neuritic plaques and neurofibrillary tangles, as well as decline in episodic memory function (Benitez et al., 2013). Another gene identified through this analysis is Doublecortin Domain Containing 2 (DCDC2), which has been suggested as a novel predictor of memory maintenance among APOE-ϵ4 noncarriers (Gao W. et al., 2022).

Associations with the progression of cognitive decline have been explored, pointing to the Dlgap2 gene as a positional candidate that modifies working memory decline (Ouellette et al., 2020). In addition, novel genetic loci associated with verbal short-term memory (VSM), learning (Lahti et al., 2022) and residual cognition (White et al., 2017) have been described. Findings from ENC1, UNC5C, and TMEM106B converged to suggest a possible role in determining cognitive resilience in the aging population affected by Alzheimer's disease, stroke and other NDs (White et al., 2017). Protective factors against late-onset Alzheimer's disease (LOAD) (Benitez et al., 2013) and risk factors associated with susceptibility to age-related cognitive decline in African Americans have also been identified. Notably, the genetic architecture of this decline appears largely similar between African American individuals and those of European ancestry (Raj et al., 2017). In the field of metabolic impairment, the PPP4R3A gene has been associated with a lower probability of metabolic decline (Christopher et al., 2017).

ROSMAP has also been instrumental in exploring associations with other psychiatric and neurological illnesses, such as depression (Demirkan et al., 2016), schizophrenia (Dobbyn et al., 2018), cerebrovascular events, Lewy bodies (LB), and hippocampal sclerosis (Farfel et al., 2016). Significant associations were identified between WWOX gene variants and neuropathological changes characteristic of limbic-predominant age-related TDP-43 encephalopathy (LATE-NC), hippocampal sclerosis (HS) and cerebral arteriolosclerosis. In addition, when exploring associations of the WWOX variant suggestive of HS, a connection was found between the rs55751884 variant and neuropathological endophenotypes, as well as with neuritic plaques (Dugan et al., 2022).

Approximately 30% of older adults are known to have the neuropathological features of Alzheimer's disease without signs of cognitive impairment. A GWAS study using data from the A4 Study, ADNI, ROSMAP, and the Adult Changes in Thought Study (ACT) performed a genome-wide analysis of AD resistance to identify biological pathways that may protect the brain from the downstream consequences of amyloidosis. The results implicate genetic drivers of bile acid homeostasis, vascular and metabolic risk factors, and neuropsychiatric conditions in Alzheimer's disease resilience (Dumitrescu et al., 2020).

Other associated characteristics have been studied, such as differences in disease course by sex (Deming et al., 2018; Eissman et al., 2022), aging and all-cause mortality (Walter et al., 2011), handgrip and lower body strength (Matteini et al., 2016), sleep duration and sleep characteristics (Lim et al., 2012), executive function and processing speed (Ibrahim-Verbaas et al., 2016), educational attainment (Okbay et al., 2016), and even associations with physical height (Yengo et al., 2022). Derived from these association studies, tools such as genetic risk scores (Chouraki et al., 2016), and new functional and scalable statistical methods for genome-wide variant modeling were developed (Chen et al., 2022).

### 2.2 High hroughput sequencing data genomics

In ROSMAP, high throughput sequencing data has been recently incorporated. Initially, sequencing of specific regions of the genome, including codons 112 and 158 of exon 4 of the APOE gene was carried out to analyze whether APOE genotype and pathological changes in the brain that occur before symptoms of AD dementia manifest could provide information on how genes contribute to the disease. Results allowed an association between APOE susceptibility alleles and intermediate neuropathological genotypes to be established. This finding was integrated in a complementary manner to other approaches, such as the consideration of factors like copy number variants (Bennett et al., 2009). Also, it was investigated how the 5 hmC marker is distributed at specific genome sites in brains of individuals with AD dementia by sequencing at specific loci (Zhao et al., 2017). WGS (whole genome sequencing) data was later generated from brain tissue (*n* = 806), whole blood (*n* = 389) and EBV-transformed lymphocytes (*n* = 5). More than 1,200 unique deceased participants have been sequenced, allowing the identification of nuclear and mitochondrial variants that affect aging-related phenotypes (De Jager et al., 2018).

Whole genome sequencing (WGS) data from the Dorsolateral Prefrontal Cortex (DLPFC), posterior cingulate cortex (PCC), and cerebellum (CB) of different individuals was used to profile mitochondrial DNA (mtDNA) copy number levels and mtDNA heteroplasmy in *n* = 762 ROSMAP brain samples. This study found that lower mitochondrial count DNA (mtcnDNA) was associated with lower cognitive performance and a more pronounced rate of cognitive decline. These results are consistent with the idea that high mtcnDNA is a feature of healthy mitochondrial function in the aging brain, suggesting that mtcnDNA may be a biomarker of aging. Furthermore, the study found that the decline in mtcnDNA is driven by certain pathologies rather than by aging *per se*, and that this decline is limited to brain regions directly affected by the respective pathology (Klein et al., 2021). Also, in 2022, WGS data was used to identify 3,012 copy number variants (CNVs) specific for AD. These genes were associated with cellular glucuronidation processes, neuronal projection, and are potential genetic regulators of the immune response in AD (Ming et al., 2022).

He et al. (2021) conducted a comprehensive exome analysis on a large dataset of AD patients. They focused on variants that influence the age of disease onset. The results of the study showed that variants in the ERN1 and SPPL2C genes are important in determining the age of onset of AD.

## 3 Transcriptomics

Transcription is a highly regulated process that ensures the adequate gene expression at the appropriate times and context (Lowe et al., 2017). Technologies such as RNA-seq allow the quantification of the presence of mRNA in biological samples, providing a view of the expression levels that characterize particular phenotypes. Through the exploration of gene expression, it is possible to study the impact of environmental factors on specific genes, the involvement of intra- or extracellular stimuli in gene expression, and the identification of regulatory elements such as promoters or repressors, among other intriguing questions (Botero and Arias, 2018).

**Figure 4:**
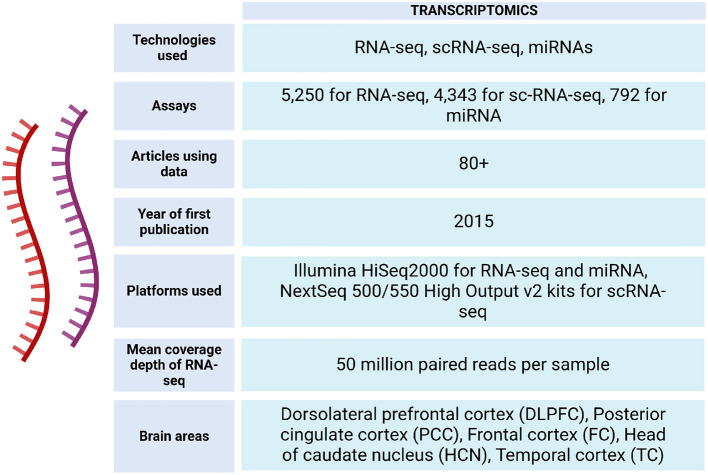
Transcriptomic data metrics in ROSMAP. For RNA-seq, sequencing was carried out using the Illumina HiSeq2000 with 101 bp paired end reads for a targeted coverage of 5 0M paired reads. Fastq files were re-aligned to the GENCODE24 (GRCh38) reference genome using STAR with twopassMode set as Basic. The RNA samples used to generate the RNAseq data were also submitted to the Broad Institute's Genomics Platform for processing on the Nanostring nCounter platform to generate miRNA profiles for 800 miRNAs using the Human V2 miRNA codeset (De Jager et al., 2018). RNA-seq data can be accessed via https://www.synapse.org/Synapse:syn26720676. For sc-RNA-seq, libraries were prepared using the Chromium Single Cell 3′ Reagent Kits v2 and the generated libraries were sequenced using NextSeq 500/550 High Output v2 kits (150 cycles). Gene counts were obtained by aligning reads to the hg38 genome (Mathys et al., 2019).

**Figure 5:**
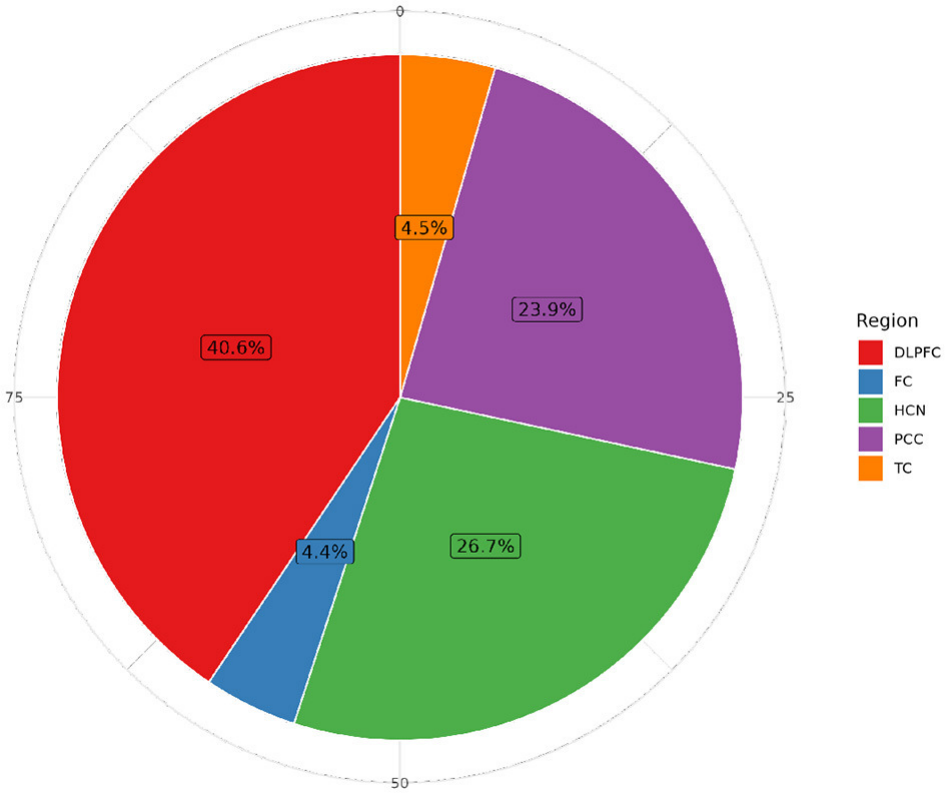
Distribution of individuals with RNA-seq sequencing data by brain tissue in ROSMAP. The Dorsolateral Prefrontal Cortex (DLPFC) is the most extensively studied region, with 1,141 specimens, followed by the Head of the Caudate Nucleus (HCN) (749 samples), the Posterior Cingulate Cortex (PCC) with 671 samples, the Temporal Cortex (TC) with 125 samples, and the Frontal Cortex (FC) with 123 samples in more than 60,607 features.

The ROSMAP project has facilitated the development of studies with different approaches, providing information from ~3,196 RNA-seq assays from the participating individuals (https://www.synapse.org/#!Synapse:syn338856) (see Figures 4, 5).

One of the broadest approaches that ROSMAP has allowed to investigate has been the identification of genes related to AD or cognitive impairment. It has been possible to identify differentially expressed genes in various cell types (excitatory and inhibitory neurons, astrocytes, oligodendrocytes, microglia, oligodendrocyte progenitor cells, endothelial cells and pericytes), which when examined in more detail have shown greater differences in early AD, suggesting that during the onset of disease progression, important transcriptional changes occur affecting all of the above cell types (Mathys et al., 2019; Barbash et al., 2017).

It has been found an association between overexpression of PADI2, ZNF385A, PSD2, and A2ML1 genes with faster cognitive decline compared to brains where they are not overexpressed (Yu et al., 2017). Also, increased expression of BACE1 in the neurons is associated with AD. It has thus been argued that BACE1 contributes to the development of pathology and symptoms associated with the disease (Li et al., 2019).

Another approach that has yielded notable results is the integration of transcriptomic data with other types of technology or comparisons with other human and mouse databases for a more complete study of the disease. The joint study of methylation and RNA-seq allowed scientists to identify that methylation of SORL1 and ABCA7 led to a change in their expression, which in turn was associated with the density of tau neurofibrillary tangles, while methylation of BIN1 and its expression were associated with Aβ burden in AD patients (Yu et al., 2015). Using ChIP-seq and RNA-seq data, H3K27ac protein was found to decrease with age in the brain (particularly in frontal and temporal regions) and was associated with increased expression of inflammatory genes such as: IL-6, TNF-αandIL-1β (Cheng et al., 2018). Furthermore, by bringing together molecular and neuroimaging data, a relationship was identified between the expression of hundreds of genes and the methylation of thousands of loci with the microstructure of specific regions in the same set of brains, i.e., a co-variation was found between these two omics and brain structure (Gaiteri et al., 2019).

A study was conducted to illustrate the utility of combining gene expression data with GWAS to investigate regulatory mechanisms of genes that may contribute to complex human traits, such as SV2A, which was found to be associated with neuroticism or anxiety (Jurkiewicz et al., 2020). It was found that increased expression in the prefrontal cortex of VEGF, FLT 4, FLT1, and PGF is associated with worse cognitive trajectories and is positively regulated in participants with AD (Mahoney et al., 2021).

### 3.1 miRNA studies

miRNA studies have also allowed the discovery of a neuroprotective pathway mediated by the coordinated negative regulation of miR-212 and miR-23a that causes a positive regulation of the SIRT1 protein which protects neurons from β-amyloid toxicity (Weinberg et al., 2015). Such studies have also allowed the comparison of differentially expressed genes found in aged mouse brains with human data, supporting the upregulation of seven of Serpine1, Plau, Hmox1, Pgf, Slc16a3, Eif4ebp1, and Lgals3 genes associated with angiogenesis and hypoxia and suggesting that changes in these genes are linked to abnormal accumulation of tau protein (Bennett R. E. et al., 2018). Studies have elucidated the role of miRNA-200 and its target genes in Alzheimer's disease (Patrick et al., 2017).

### 3.2 Transcriptomic networks

Also, based on transcriptomic data, gene co-expression networks have been constructed for males and females to identify co-expressed AD-associated gene modules that are shared or sex-specific. Here, LRP10 was identified as a major driver of sex differences in AD pathogenesis and manifestation, and experiments were performed in mice to demonstrate its role. LRP10 was found to differentially affect cognitive function and AD pathology as a function of sex and APOE genotype (Guo et al., 2023).

On the other hand, AD has also been approached from network science, studying it as the result of the complex interaction of multiple genes, rather than attributing it to a single (or a few) genetic abnormalities (Chen et al., 2019). This approach has allowed scientists to identify changes in the activity of groups of genes associated with different functions during the disease, such as synaptic signaling, metabolism, cell cycle, survival and immune response (Canchi et al., 2019).

Other approaches that have been explored with the use of transcriptomics are the correlation of some diseases and their relationship to the development of AD.

For example, the relationship between type 2 diabetes (DT2) and Alzheimer's dementia was explored by identifying 13 common causal genes, 16 common causal pathways, as well as 754 gene expression nodes and 101 gene methylation nodes associated with both AD and DT2 in multi-omics causal networks (Hu et al., 2020). Similarly, the effects of bile salt metabolism and bile production, as well as their role in the development of AD have been explored (Varma et al., 2021).

Neuroticism has also been implicated as a factor that alters the transcriptome of the DLPFC and contributes to the development of cognitive impairment and Alzheimer's dementia (De Jager et al., 2018). Even in cerebral amyloid angiopathy, the rs28660566T variant in the UNC5C gene was found to be associated with a higher pathology score. However, it was weakly associated with lower UNC5C expression in the DLPFC, and no association with disease severity was found (Yang et al., 2017). Concerning Parkinson's disease, it has been suggested that age and the H2 variant of the MAPT gene are associated with an increased risk, as well as lower overall MAPT expression (Valenca et al., 2016). Although overall inflammatory disease risk does not appear to have a significant impact on age-related cognitive decline, genetic variants associated with diseases such as multiple sclerosis (MS), coronary artery disease (CAD) and rheumatoid arthritis (RA) that affect peripheral immune function also alter microglial density and immune gene expression in the aging brain (Felsky et al., 2018). Also, the relationship between prolonged periods of sleep deprivation, the activation of astrocyte activator genes, and how this activation conditions cognitive impairment and AD have been explored (Kaneshwaran et al., 2019).

Biological processes such as diet, sleep and even change of seasons have also been studied and found to be associated with the development of AD. Li et al. (2019) conducted a study using RNA sequencing where it was found that healthy diets can positively regulate the expression of TCIM and MPO genes, which have been related to educational attainment and cognitive performance in depression in previous GWAS (Okbay et al., 2022; Thalamuthu et al., 2022). Moreover, the CC2D2B, PDXDC2P, and MBIP genes exhibited strong negative associations, which are also linked to Alzheimer's disease (AD) (Gouveia et al., 2022), thus suggesting that healthy diets may help maintain cognition during aging. Angiogenesis factors such as NRP1, VEGA, VEGFB, and FLT are predisposing factor for the development of AD. Transcriptomics has enabled us to observe interactions between these conditions. For example, angiogenesis regulatory genes such as NRP1 and VEGA were found to interact with APOE-ϵ4, as patients carrying APOE-ϵ4 with higher NRP1 expression have worse outcomes compared to patients not carrying the allele. The role of angiogenesis-related genes as part of the pathophysiology of AD was also observed in a study by Seto et al. (2023), who used transcriptomic technologies to characterize brain changes in the vascular endothelial growth factor family during aging and AD, found that VEGFB and FLT1 expression were associated with worse outcomes, and that microglia, oligodendrocytes and endothelial may play a central role in these associations. Differential gene expression analysis between AD patients and controls was performed in both blood and brain samples using a multivariate approach, both in the total sample and in the subgroup with APOE genotypes. The results suggest an influence of APOE genotype on the configuration of expression network profiles in both blood and brain. Several genes belonging to these networks were found to be associated with markers of vascular injury, possibly contributing to the effect of the ϵ4 allele on the integrity of the blood-brain barrier (BBB) (Panitch et al., 2022).

## 4 Proteomics

Proteomics focuses on the study of protein composition, structure, function and interactions (Al-Amrani et al., 2021). Proteins represent an important source of pharmacological molecular targets, and the information gathered about them may be essential for the development of new therapies and drugs (Robins et al., 2021). While proteomics represents a relatively recent addition to the omics field, it possesses inherent limitations. Notably, proteomic techniques do not directly measure protein expression, but rather infer the abundance of known protein isoforms based on the employed methodology. This indirect approach arises from the challenge of quantifying protein expression due to the complex and often inconsistent relationship between mRNA levels and their corresponding canonical proteins (Carbonara et al., 2021). Despite this, proteomics has had a rapid development thanks to its therapeutic applications, such as the identification and monitoring of pathological biomarkers and the development of new drugs (He and Chiu, 2003).

**Figure 6:**
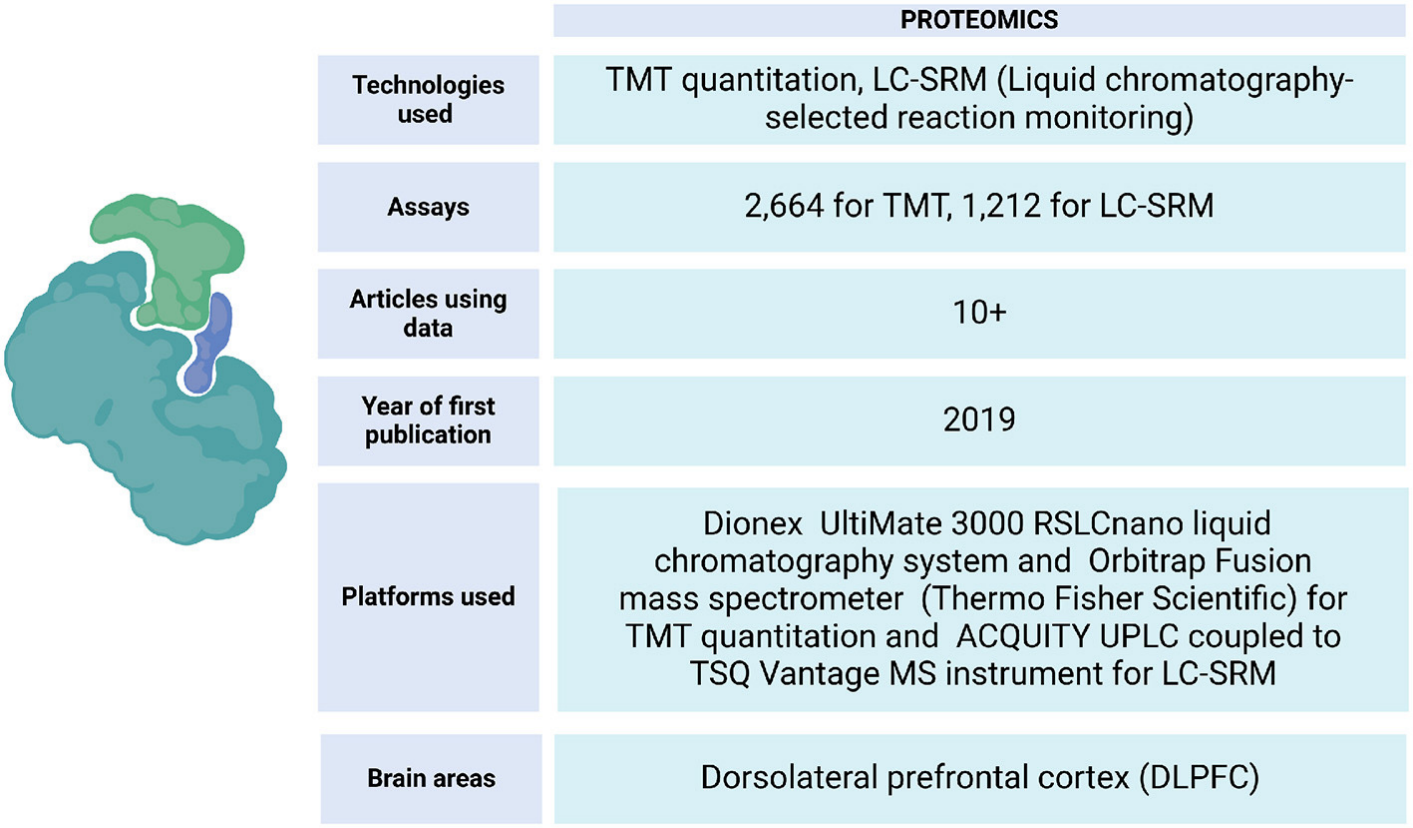
Proteomic data metrics in ROSMAP. LC-SRM and TMT-MS proteomics was performed using frozen tissue from dorsolateral prefrontal cortex (DLPFC). In LC-SRM, the abundance of endogenous peptides was quantified as a ratio to spiked-in synthetic peptides containing stable heavy isotopes. For TMT, MS2 spectra were searched against the UniProtKB human proteome database containing both Swiss-Prot and TrEMBL human reference protein sequences (90,411 target sequences), plus 245 contaminant proteins. Both TMT quantitation and LC-SRM data can be accessed via https://www.synapse.org/Synapse:syn17008935.

ROSMAP has data from protein expression quantifications of the DLPFC and other brain tissues. These samples have been processed by liquid chromatography-selected reaction monitoring (LC-SRM) and tandem mass labeling (TMT) (Johnson et al., 2020) (Figure 6). This data has allowed the identification of correlations between the influence of genetic variation with mRNA and protein abundance on the Alzheimer's disease phenotype (Roberts et al., 2021), identification of changes in the expression of specific proteins in brain regions of cognitive importance, such as the prefrontal and temporal cortices (Johnson et al., 2022), linking post-translational modifications that are related to the progression of AD (Dammer et al., 2022), localizing specific brain regions with increased vulnerability to the disease (Hsieh et al., 2019), building protein association studies integrated with QTLs, and identifying brain proteins that present evidence consistent with being causal in AD (Wingo et al., 2021). Also, an analysis strategy to map resilience-associated pathways and extend mechanistic validation was performed. Proteomic data was generated from Brodmann areas 6 and 37 of brain tissue and analyzed using consensus-weighted gene correlation network analysis (WGCNA). Notably, neuritin (NRN1), a neurotrophic factor previously implicated in cognitive resilience, emerged as a core protein within a module linked to synaptic biology. Functional experiments in a cellular model of AD revealed that NRN1 protected dendritic spines from amyloid-β (Aβ) toxicity and suppressed Aβ-induced neuronal hyperexcitability (Hurst et al., 2023).

Intending to identify loci conferring AD risk through their effects on brain protein abundances, results from AD GWAS were integrated with human brain proteomes to perform a whole proteome association study (PWAS) of AD, followed by Mendelian randomization and colocalization analysis. Eleven APOE4-independent genes consistent with being causal in AD were identified, acting through their cis-regulated brain protein abundances. Nine were replicated in a confirmatory PWAS and eight represent novel AD risk genes not previously identified by AD GWAS (Wingo et al., 2021)

Another study, aiming to expand the known causal proteins for Parkinson's disease (PD), used PWAS data from human brain proteomes of DLPFC and applied a systematic pipeline through multi-omics analysis. It described that GPNMB showed a genetically causal role for PD, and that DGKQ and CD38 may have a protective function. Causally related proteins were found in blood and in the cerebrospinal fluid (CSF). This study suggested that GPNMB, CD38, and DGKQ may act in the pathogenesis of PD (Gu et al., 2023).

Pathak et al. (2022) employed mRNA integration, alternative splicing analysis, and proteomic profiling of the DLPFC to characterize the biological heterogeneity of post-traumatic stress disorder (PTSD) symptom clusters. Using a systems-biology approach, their investigation identified genes associated with specific PTSD symptoms—reexperiencing, hyperarousal and avoidance—presentations of the disease. They used three regulatory models—TWAS, SPWAS, and PWAS—resulting in the identification of 30 unique gene associations at 19 independent genomic regions across the three PTSD symptom clusters. Seven genes (KHK, CGREF1, RBM6, MAPT, CRHR1, RNF123, ARHGAP27) were common to all the three PTSD symptoms, while one (RBMX1), seven (EXOC6, CDC14B, CTNND1, SERGEF, CEP57, WNT2B, B3GALTL), and nine (HARS2, PDLIM2, TSFM, RAB27B, MAPRE3, NDUFA2, PCDHA7, TPM3, NCK1) were distinct to Reexperiencing, Hyperarousal and Avoidance, respectively.

Studies using proteomic technologies have contributed significantly to our understanding of neurodegenerative diseases and cognitive aging, providing a clearer picture of the biological processes involved. These findings have important implications for diagnosis, patient stratification, and the development of targeted therapies for conditions such as AD and mild cognitive impairment (Roberts et al., 2021). Collectively, these experiments highlight the value of integrating human brain proteomic data with model systems. This integrated approach has the potential to help elucidate the mechanisms that contribute to brain resilience in the face of pathology and ultimately guide the prioritization of therapeutic interventions.

## 5 Epigenetics

Epigenomics refers to the collection and study of chemical and protein modifications that act upon DNA to regulate gene expression without modifying the underlying genetic sequences (Kato, 2022). Histone modifications, non-coding RNA regulation, chromatin remodeling, and DNA methylation are the main areas of current understanding regarding the role of epigenetics in the mechanism of AD (Liu et al., 2018; Kundaje et al., 2015).

**Figure 7:**
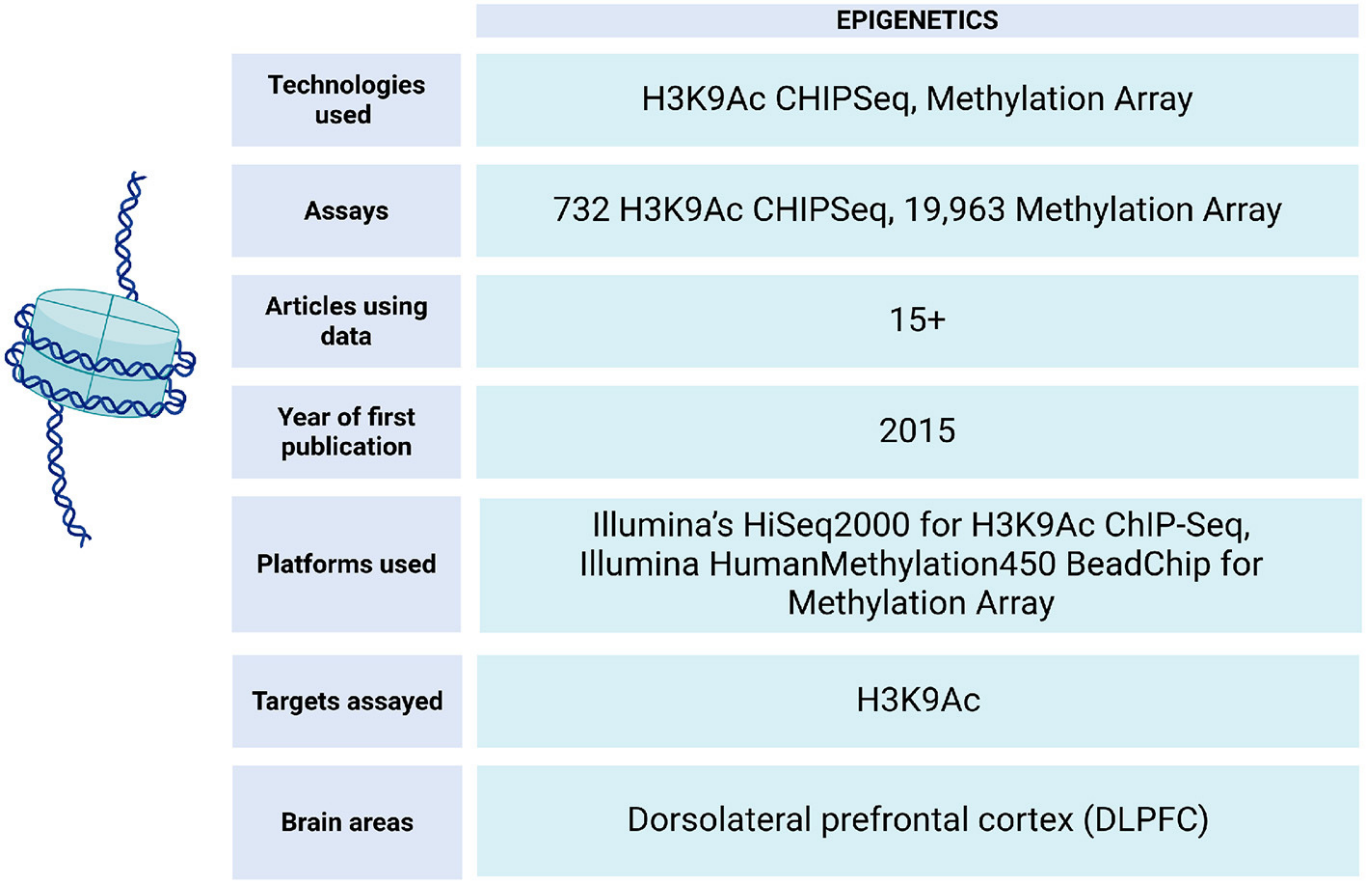
Epigenetic data metrics in ROSMAP. For H3K9Ac ChIP-Seq, the Millipore anti-H3K9Ac mAb was used for chromatin immunoprecipitation experiment can be found in Synapse:syn4896408. H3K9Ac ChIP-Seq Methylation data can be found in Synapse:syn3157275.

In ROSMAP, epigenetic data is obtained through ChIP-seq experiments (Klein et al., 2021) and Illumina HumanMethylation450 BeadChip technology (De Jager et al., 2014) (Figure 7). This data has enabled epigenetic clock analysis, a mathematical tool that links DNA methylation patterns with the chronological age of individuals. This approach makes it possible to estimate the biological aging of an individual based on epigenetic patterns, which provides valuable information about his or her health and aging process (Grodstein et al., 2021; Horvath et al., 2016).

The study led by Grodstein et al. (2021) provides a view of the relationship between DNA methylation and brain aging. It highlights the importance of considering common neuropathologies when analyzing age-related epigenetic modifications. Although more than half of the participants received a diagnosis of AD, no correlation was found between epigenetic clock age and conditions such as atherosclerosis, arteriolosclerosis, or cerebral amyloid angiopathy. This finding emphasizes the relevance of adjusting analyses for the presence of common neuropathologies when exploring associations between DNA methylation and age, as evidenced in an earlier study (Yang et al., 2015). It was also shown that adjustment for common neuropathologies significantly reduced the number of age-related CpG sites, and variability in the direction of the associations was observed, with most of the significant sites being hypomethylated.

On the other hand, the study conducted by Thrush et al. (2022) used DNA methylation data from 450 K and EPIC arrays, excluding CpG on sex chromosomes, to develop a multi-region methylation clock that estimated brain age in the context of AD. The brain age predictor demonstrated a strong correlation with chronological age and provided accurate information in multiple brain regions, revealing potential as a biomarker of AD. In addition, gene set enrichment analyses (GSEA) identified CpG and related genes, providing insights into the molecular mechanisms underlying brain aging and AD.

Taken together, these findings contribute to the understanding of epigenetic dynamics in the brain aging process and reinforce the need to consider the influence of common neuropathologies when interpreting age-related epigenetic modifications in the human brain. Furthermore, they highlight the utility of methylation-based clocks in understanding the relationship between brain aging and AD.

In an effort to gain insight into the interactions between methylation and transcriptomic and proteomic networks in AD, 270 differentially methylated regions were identified in postmortem AD-associated brains. The results revealed that methylation significantly impacts AD-associated gene/protein modules and their key regulators, such as TNPO1. This suggests that methylation plays a crucial role in the regulation of primary network drivers and their downstream genes (Wang et al., 2023).

Other Epigenome-Wide Association Study (EWAS) using ROSMAP found 130 CpGs (including 57 novel ones) and twelve genetic regions, such as ANK1 and BIN1, significantly associated with amyloid burden. DNA methylation in some regions was linked to gene expression and positive correlations were observed between neuropathological burden, age, gender and educational level. DNA methylation in the BIN1, SPG7, RHBDF2, and GMDS genes was positively associated with their expression, whereas DNA methylation in the PODXL gene was inversely associated with their expression (Palma-Gudiel et al., 2023). On the other hand, a region of hypermethylation in the prefrontal cortex and superior temporal gyrus associated with AD was identified. This region, which encompassed the HOXA gene cluster, showed consistency across different cohorts. Braak's meta-stage analysis revealed increased DNA methylation in the prefrontal cortex. In addition, a significant correlation was found between methylation in the HOXA gene region and the ANK1 gene, suggesting a key role of hypermethylation in AD progression (Smith et al., 2018).

In addition, the integration of genetic, epigenetic and transcriptional data has allowed the identification of components involved in cognitive resilience, such as UNC5C, ENC1, and TMEM106B (White et al., 2017). It has also contributed to the development of a multi-omics atlas of the parahippocampal gyrus in AD, ranging from whole genome sequencing to cell type-specific RNA and ATAC-seq data. These data are intended to drive the development of new therapies and biomarkers for AD by providing a public resource on the Synapse platform for the research community. The integration of multi-omics data reveals detailed signaling maps of regulatory cascades in AD, highlighting associations between DNA methylation, chromatin accessibility, transcription and translation. Furthermore, AD-associated methylomic changes, differentially methylated regions (DMRs) correlated with gene and protein expression levels were identified, and a global methylation score was developed to quantify their impact on individual genes and proteins. Causal inference evidence suggests that DMRs influence gene/protein expression through ATAC peak domains in AD (Coleman et al., 2023).

In summary, studies conducted within the framework of ROSMAP and other EWAS have provided valuable contributions to understanding the complex relationship between DNA methylation, brain aging and AD. Taken together, these findings contribute to the formation of a more comprehensive understanding of epigenetic dynamics in brain aging and neuropathological processes, underscoring the importance of interdisciplinary approaches to address the challenges of neuro-epigenetic research.

## 6 Metabolomics

Metabolomic profiling offers significant potential for uncovering dysregulations in biochemical pathways linked to dementias. Increasing evidence indicates that these dementias may result from underlying metabolic abnormalities (Horgusluoglu et al., 2022; Varma et al., 2021).

**Figure 8:**
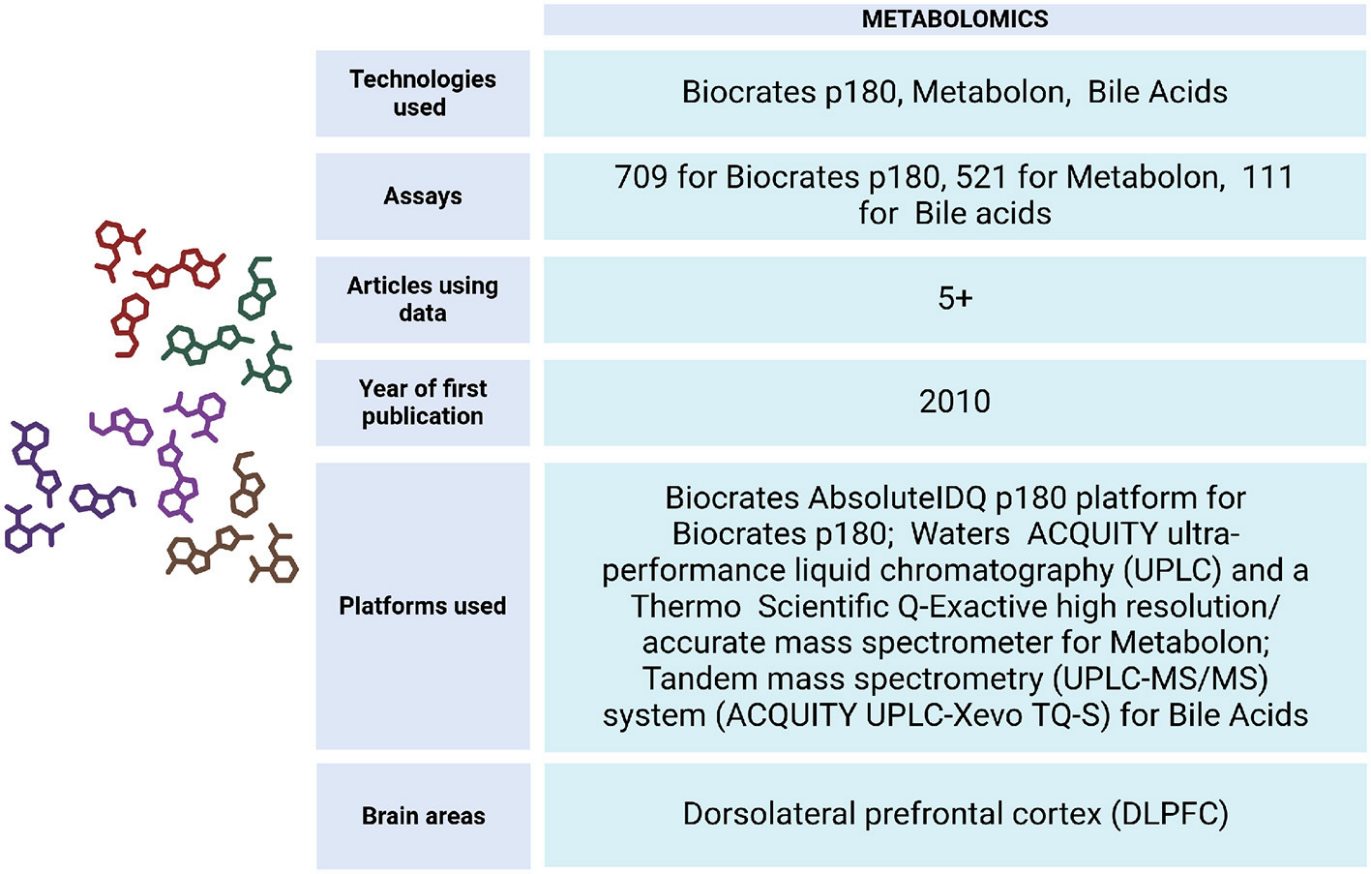
Metabolomics data metrics for ROSMAP. The Biocrates AbsoluteIDQ p180 platform (Biocrates AG, Innsbruck, Austria) was used for this Biocrates p180 assay. It is a multiplexed targeted metabolomic assay covering 188 metabolites, including hexoses, amino acids, biogenic amines, acylcarnitines, glycerophospholipids and sphinoglipids. An ultra-performance liquid chromatography couple to tandem mass spectrometry (UPLC-MS/MS) system (ACQUITY UPLC-Xevo TQ-S, Waters Corp., Milford, MA) was used to quantitate bile acids. Metabolon assay methods utilized a Waters ACQUITY ultra-performance liquid chromatography (UPLC) and a Thermo Scientific Q-Exactive high resolution/accurate mass spectrometer interfaced with a heated electrospray ionization (HESI-II) source and Orbitrap mass analyzer operated at 35,000 mass resolution. Metabolomics data can be accessed via https://www.synapse.org/Synapse:syn10235592.

To understand the metabolic changes that occur during diseases of aging, the ROSMAP project has generated data from DLPFC tissue and serum samples (Figure 8). These include Biocrates p180 assays, which analyze metabolites from various classes such as hexoses, amino acids, biogenic amines, acylcarnitines, glycerophospholipids, and sphingolipids. Additionally, Metabolon HD4 assays, UHawaii Bile Acids assays, mass spectrometry (Morgenstern et al., 2022), ultra-performance liquid chromatography triple quadrupole mass spectrometry (UPLC-TQMS), and gas chromatography time-of-flight mass spectrometry (GC-TOFMS) have been conducted on both living and non-living individuals (Wang et al., 2020).

In a diagnostic evaluation of F2-isoprostane as a potential biomarker for dementia *in vivo*, isoprostane levels were quantified in plasma and urine samples obtained from 222 participants encompassing a spectrum of cognitive health, including healthy controls and individuals with established dementia diagnoses. Gas chromatography-mass spectrometry (GC-MS) served as the analytical method to assess these levels. Notably, the findings did not reveal a significant discriminatory power of plasma or urinary isoprostane levels in differentiating between individuals with and without cognitive impairment (Mufson and Leurgans, 2010). Furthermore, to characterize the metabolic landscape and its association with neuropathology and cognitive function, metabolic profiling was employed in both brain and matched serum samples. The abundances of six metabolites, glycolithocholate (GLCA), petroselinic acid, linoleic acid, myristic acid, palmitic acid, palmitoleic acid and the deoxycholate/cholate (DCA/CA) ratio, along with the dysregulation scores of three metabolic pathways, primary bile acid biosynthesis, fatty acid biosynthesis, and biosynthesis of unsaturated fatty acids showed significant differences across both brain and serum diagnostic groups. Moreover, the identified metabolite abundances and personalized metabolic pathway scores were leveraged to construct machine learning models capable of differentiating individuals with and without cognitive impairment. This approach underscores the potential of metabolomics as a diagnostic tool for cognitive decline (Wang et al., 2020).

The progression of AD is associated with low levels of short-chain acylcarnitines, as well as several amines and amino acids, whereas there is a strong correlation between high levels of medium-chain acylcarnitines and the composite memory score on the Mini-Mental State Exam. In a study that used ROSMAP as replication cohort, researcher showed acylcarnitines and amines are highly correlated with AD clinical outcomes and further reveal key biological drivers and pathways that are involved in metabolomic changes in mild cognitive impairment and AD (Horgusluoglu et al., 2022).

## 7 Multiomics

**Figure 9:**
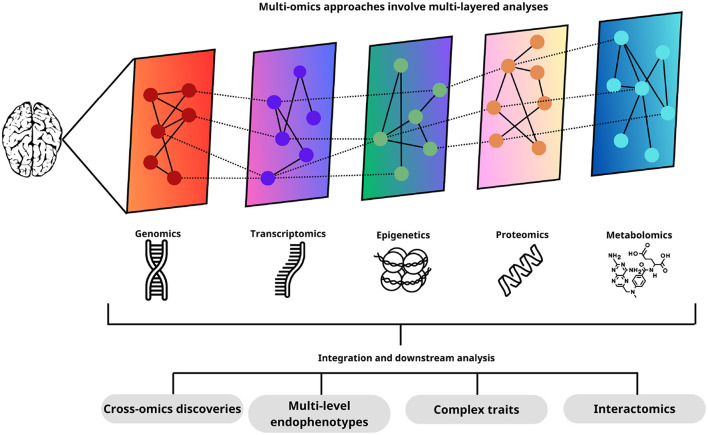
Each of the omics layers provides a distinctive set of data that, when integrated and analyzed together, allows a deep understanding of biological processes. This integrated approach facilitates the identification of new interactions between different omics levels, the discovery of endophenotypes at multiple levels, the elucidation of complex traits, and the study of interactomics. Thus, multi-omics analyses provide a holistic and comprehensive view of brain biology, opening new avenues for biomedical research.

The independent analysis of different types of omic data is often restrictive for the detection of consistent variations in different levels of information with explanatory potential between them. Multiomics studies explore the multidimensional interactions between various levels of information from omics technologies, offering a holistic perspective of biological processes Figure 9. These studies enable the elucidation of complex system descriptions and facilitate the advancement of medical and biological knowledge (Song et al., 2020).

The interplay between various layers of molecular regulation, encompassing genetics, epigenetics, mRNA expression (Tasaki et al., 2022), proteomics (Xie et al., 2020), and metabolomics (Iturria-Medina et al., 2022) collectively contributes to the pathogenesis of age-related neurological disorders (Figure 8). Multiomics data has the potential to more accurately represent the traits of AD patients from a variety of uncorrelated angles when compared to single data (Gao Y. et al., 2022). A key strength of ROSMAP omics data lies in its versatility for integration across different levels and modalities. This potential has been demonstrated in numerous studies that leverage data from multiple omics technologies. For instance, research aimed at identifying novel therapeutic targets and compounds for AD treatment and prevention has employed integrative strategies. These strategies combine disciplines such as systems biology, proteomics, and functional validation alongside molecular screening techniques, all while utilizing diverse omics data types (Bennett et al., 2014).

Also, through a multistep analysis of clinico-cognitive, neuropathological, genomic, epigenomic and transcriptomic data, genes related to the dissociation of cognition and neuropathology have been identified. For example ENC1, UNC5C, and TMEM106B have been suggested as determinants of cognitive resilience in the aging population affected by Alzheimer's disease, stroke and other neuropathologies (White et al., 2017). To better understand the specific effects of the APOE gene in the pathogenesis of AD, Madrid et al. (2021) analyzed and integrated publicly available data from multiple omics technologies from both plasma and brain stratified by APOE haplotype (APOE2, APOE3, and APOE4). Combining genome-wide association studies (GWAS) with differential analyses of protein and mRNA expression and single core transcriptomics from multiple cohorts that included ROSMAP, genes and pathways that contribute to AD in both an APOE-dependent and APOE-independent manner were found. A study using DNA methylation and RNA-seq suggested that epigenetically regulated expression of the MGMT (Methylated-DNA-protein-cysteine methyltransferase) gene, involved in DNA damage repair function, is significantly associated with the development of the hallmark AD proteins amyloid-β and tau, especially in women (Chung et al., 2023).

The functional relevance of results from a genome-wide association study (GWAS) of verbal declarative memory (VDM) has also been analyzed by integrating multi-omics data from twenty-seven cohorts comprising individuals of Caucasian descent. The results add to the growing evidence implicating the regulation of expression, immunity, and insulin deficiency in memory impairment (Mei et al., 2024).

Mei et al. (2024) conducted a comprehensive analysis of how genetic variations identified by genome-wide association studies (GWAS) affect verbal memory. Delayed verbal declarative memory (VDM) performance is a key predictor of Alzheimer's disease (AD). The study aimed to discover new strategies for treating or preventing memory loss in older adults with dementia. To achieve this, the researchers analyzed and integrated multi-omics data from twenty-seven cohorts of individuals of Caucasian descent. Nineteen of these cohorts included individuals without dementia or stroke, while the remaining eight included participants without stroke, but their dementia status was not available.

The study found that genetic associations with VDM influence the regulation of gene expression through expression quantitative trait loci (eQTL) and methylation quantitative trait loci (meQTL). These genetic variations may affect gene expression in the brain, impacting memory performance and the risk of developing Alzheimer's disease, particularly through immune mechanisms.

### 7.1 Multiomics network analysis in systems biology

In recent years, the use of systems biology with multiomics approaches has been implemented to address diseases of aging, for example, by inferring interactions between regulatory elements capturing genetic and epigenetic influences on expression in older adults. One of the most widely used approaches has been biological networks. In this context, predictions using local regulatory networks (LRNs) have identified a specific set of largely neuronal genes, such as STAU1 and SEMA3F, that are predicted to control cognitive decline in older adults (Tasaki et al., 2018). In another work, a constrained modularity model was proposed to jointly analyze genotype, gene expression and protein expression data, which was tested on ROSMAP multiomics data, identifying a functionally connected subnetwork including 276 multiomics biomarkers, including SNPs, genes and proteins. The results suggest that impaired cognitive performance in AD patients may potentially be the result of genetic variations due to their cascading effect on the transcriptome and proteome (Xie et al., 2020). Another work with a network approach in which ROSMAP was used as a replication cohort. Probabilistic causal models have been also constructed that allowed the detection, prioritization and replication of high-confidence master regulators of AD-associated networks, including the predicted master regulator, the neuropeptide VGF (VGF nerve growth factor inducible), which was also validated in murine models. These findings support a causal role of VGF in protection against AD pathogenesis and progression (Beckmann et al., 2020).

By employing a coexpression network approach in four cortical areas and subsequently integrating with animal models, researchers have identified critical gene subnetworks associated with late-onset Alzheimer's disease (LOAD). ATP6V1A has been proposed as a key regulator within a specific neuronal subnetwork particularly affected by this disorder (Wang et al., 2021). Another investigation employing rigorous multi-step validation has yielded consistent findings. This work demonstrates global deregulation of the sphingomyelin pathway at the gene expression level across all brain regions examined in AD patient samples. Notably, the expression of 20 out of 35 genes encoding key enzymes within this pathway was found to be significantly dysregulated in the AD population (Baloni et al., 2022). Other works have also explored regulation and co-expression networks to evaluate cerebrovascular effects of APOE genotypes (Panitch et al., 2022), and identification of master transcription factors in AD, where possible key factors in Alzheimer's disease have been discovered, such as the JMJD6 gene, which was also validated in an animal model (Merchant et al., 2023).

In microglia, marker genes such as CEBPB, STAT3, and SPI1 have been identified, which could act as mediators in the alterations in gene regulation associated with AD, exerting a significant impact on its pathogenesis. This work was conducted by analyzing single-nucleus RNA sequencing (snRNA-seq) and bulk brain RNA sequencing (RNA-seq). Using this approach, immunotherapy targeting drug-core transcription factors (TFs) was found to be significantly different among AD patients (Gao W. et al., 2022).

Molecular subtyping of brain tissue enables a better understanding of the heterogeneity in neurodegenerative diseases. To perform molecular brain tissue ad subtyping by multiple analyses, DLPFC samples were analyzed at five levels, including RNA-seq, DNA methylation, histone acetylation, proteomics and metabolomics to which a fusion of unsupervised similarity networks was applied. This is a method capable of simultaneously integrating multiple modalities of high-dimensional multiomics data. This study supports that expression (RNAseq) and epigenetic (H3K9ac) patterns demonstrate variability patterns more aligned with cognitive decline (Yang et al., 2023). Another recent study identified genetic modules in networks (including 263 genes) that were related to the integrated aging measurement of six molecular clocks, as well as three neurological traits of AD (i.e., β-amyloid, Tau tangles and tangle density) and age. Among the 20 key genes with superior intramodular connectivity of the module, PBXIP1 was the only one that was significantly associated with the three neuropathological traits of AD at the protein level (Zhang et al., 2024).

Integration of Multiomics Data from ROSMAP with Automated Learning Models Advances in machine learning models are transforming disease classification through the analysis of molecular data, which is especially relevant in the context of complex diseases characterized by a dispersed molecular background. Effective training of these tools demands the availability of extensive and meticulously described databases. In this context, ROSMAP has been frequently used, obtaining encouraging results in the multiomics classification of both healthy and disease-affected individuals. Several approaches that use data from DNA methylation, RNA-seq, microarrays and/or miRNAs have been proposed, such as the integrative copula discrimination analysis (ICDA) which can make diagnostic predictions (He et al., 2020), or the generalizable model called Multi-Omics Graph cOnvolutional NETworks (MOGONET) that uses graph convolutional networks (GCN), which achieved the identification of mRNA, DNA methylation and miRNA expression biomarkers (Wang et al., 2021). Other tools that have used multiomics data from the cohort for classification is the Multi-task Attention Learning Algorithm for Multi-omics Data (MOMA), which vectorizes features and modules using a geometric approach and focuses on important modules in the data through an attention mechanism (Moon and Lee, 2022); DeepOmicsAE, an optimized workflow for proteomics, metabolomics and clinical data set analysis (Panizza, 2023); iNTEgrate, which integrates transcriptome and DNA methylation data into a single gene network (Sajedi et al., 2023); GREMI, which uses mRNA, methylation and miRNAs (Liang et al., 2023); an Explainable variational autoencoder (E-VAE) classifier model, which uses genome-wide SNPs and RNAseq data (Vivek, 2023); MOSEGCN, a deep learning multi-omics integration model for the classification of complex diseases (Wang et al., 2024); and TEMINET, that uses intra-omic features to build disease-specific care networks (Luo et al., 2024).

Advanced machine learning analysis models of multiomics molecular data from postmortem brain and blood *in vivo* have already been used to obtain personalized multilevel molecular indices of AD dementia progression to predict the severity of neuropathologies. For example, one study identified three robust molecular-based subtypes that explain much of the pathological and clinical heterogeneity of AD. These subtypes exhibited different profiles of alterations in the methylation of DNA, RNA, proteins and metabolites, which are detectable in both brain tissue and blood samples (Iturria-Medina et al., 2022). Another example is the work of Khullar and Wang (2023), who performed an integrative multiomics analysis to predict gene regulatory networks for three main brain regions: Hippocampus, Lateral Temporal Lobe (LTL), and DLPFC. A list of six AD-COVID relationship candidate genes was identified to predict the severity of COVID. Their ability to predict AD was evaluated, and it was demonstrated that they are also predictors of AD severity, as they outperformed their respective benchmark models and showed promising clinical potential for predicting immune dysfunction, inflammation, AD, and severe/neurological COVID.

### 7.2 Harmonization of data between multiomics cohorts of aging and neurodegenerative diseases study

Harmonization has been carried out not only between omics data, but also between cohorts with related omics information. Such is the case of The Whole Genome Sequence Harmonization Study (WGS_Harmonization) (https://adknowledgeportal.synapse.org/Explore/Studies/DetailsPage/StudyDetails?Study=syn22264775), and The RNAseq Harmonization Study (rnaSeqReprocessing) (https://adknowledgeportal.synapse.org/Explore/Studies/DetailsPage/StudyDetails?Study=syn9702085) which harmonize WGS and RNA-seq data from three longitudinal cohorts ROSMAP, MSBB and MayoClinic with aging, dementia and AD foci, giving a total of 1,796 individuals with RNA-seq data from tissues such as cerebellum, temporal cortex-frontal pole, inferior frontal gyrus, parahippocampal gyrus, prefrontal cortex, superior temporal gyrus, DLPFC, frontal cortex, head of caudate nucleus and posterior cingulate cortex, 1,872 individuals with WGS data, and 1,501 subjects with both RNA-seq and WGS data.

This synergistic approach empowers researchers to achieve more accurate, reliable, and generalizable results. It is primarily facilitated by two key aspects: a substantial increase in the number of samples accessible for investigation, and the enrichment of analyses through the inclusion of diverse tissue types. The harmonization of these data is expected to improve the confidence of the findings validity, by improving the interpretation related to missing information.

## 8 Concluding remarks

The ROSMAP initiative has significantly contributed to the understanding of Alzheimer's disease through its comprehensive integration of various omic datasets. By leveraging genomics, transcriptomics, proteomics, metabolomics, epigenomics, and multiomics, ROSMAP has given great tool to elucidate the intricate molecular interactions that occur in the context of ND and aging. Omics research, combined with clinical variables and tools such as imaging, has significantly advanced our understanding of the pathophysiological mechanisms underlying Alzheimer's disease. This integration has provided new insights into potential diagnostic markers and therapeutic targets. The extensive and longitudinal nature of the ROSMAP data highlights the importance of multi-dimensional approaches in unraveling the complexities of neurodegenerative diseases. This comprehensive strategy paves the way for more effective interventions and improved clinical outcomes for individuals affected by neurodegenerative diseases such as Alzheimer's disease, Parkinson's disease, and psychiatric conditions like PTSD, depression, anxiety, among others affecting the aged brain.

There are still various aspects that can be explored using the ROSMAP database. For example, metabolomics, a highly complex discipline, has been relatively underexplored in ROSMAP studies. This may be due to the significant technical and analytical challenges associated with studying a broad spectrum of metabolites with diverse chemical properties. Additionally, external factors like diet and lifestyle can affect metabolite concentrations, complicating result interpretation. It would be interesting to explore metabolomic data further and obtain information from other areas of the brain and different body parts in both living and deceased individuals to complement the landscape of metabolites involved in neurodegenerative diseases. Although epigenetic studies have been conducted, the interaction between epigenetics and other omics data remains underexplored. Utilizing integrative approaches to combine epigenetic data with transcriptomic and proteomic data can help identify how epigenetic modifications influence gene expression and protein production in the context of neurodegenerative diseases. The implementation of machine learning algorithms and big data techniques can facilitate this integration and help uncover new biological connections. Studies such as De Jager et al. (2014) have begun to explore these interactions, but more in-depth research is needed.

Current studies predominantly focus on genetic and biological factors, underestimating the importance of environmental and lifestyle factors. Collecting data on environmental and lifestyle factors can be laborious and costly. Moreover, these factors often require long-term longitudinal studies to be adequately assessed. The lack of resources and the necessary infrastructure to carry out these studies can be a significant limitation. Collect detailed data on participants lifestyle, diet, physical activity, and environmental exposure, and analyze the interaction between these factors and omics data. This could include the use of detailed questionnaires and wearable devices to monitor physical activity and other health parameters. Including these variables could provide a more comprehensive understanding of the multifactorial etiology of Alzheimer's disease. Studies such as Livingston et al. (2017) suggest that lifestyle and environmental factors play a crucial role in the development of Alzheimer's, indicating the need for further research in this area.

Single-cell RNA sequencing (scRNA-seq) is another approach that has recently emerged as a powerful tool for investigating cellular biology at an unprecedented resolution and is expected to have a significant impact on understanding molecular pathophenotypes. As in other cases, scRNA-seq studies have been limited to the dorsolateral prefrontal cortex (DLPFC). Limitations in accessing other brain regions and the viability of post-mortem cells may restrict studies to the DLPFC. There might also be a bias toward this region due to the availability of standardized protocols and methods for DLPFC analysis.

Expanding the use of scRNA-seq to other brain regions such as the hippocampus, amygdala, and entorhinal cortex, which are also implicated in Alzheimer's disease, is crucial. Additionally, using scRNA-seq to study cellular heterogeneity and changes in gene expression in different cell types, such as neurons, astrocytes, and microglia, across multiple brain regions could provide a more comprehensive view of neurodegenerative pathologies. Recent studies, such as those by Mathys et al. (2019) and Grubman et al. (2019) have demonstrated that scRNA-seq can reveal specific cellular subpopulations and their roles in neurodegenerative diseases, suggesting the need for further exploration in other brain regions. Further advances on single cell technologies have indeed allowed for comprehensive and highly detailed characterization of the AD molecular phenotype in more specific brain regions (Mathys et al., 2024).

However, since the majority of publicly available omics data has focused predominantly on non-Hispanic white subjects, it is crucial to address the existing disparity in research representation. Initiatives such as the Research in African American Alzheimer's Disease Initiative (REAAADI) (Akgun et al., 2022) and the Asian Cohort for Alzheimer's Disease (ACAD) (Ho et al., 2024) are emerging to conducting research within non-Hispanic white communities, focusing initially on the genetic level. Other initiatives are conducting multi-omics studies in Native American populations (Reddy et al., 2024). By expanding research to include a broader spectrum of ethnic and racial groups, we can improve the generalizability of findings and enhance the development of targeted therapeutic interventions for all populations.

The utilization of omics technologies has proven critical in the study of Alzheimer's disease and mild cognitive impairment. However, to comprehensively understand the underlying biological processes and disease mechanisms, it is imperative to integrate multi-omics data with additional datasets. This includes clinical assessments, imaging studies, cognitive performance metrics, and data from other parts of the body besides the brain, such as blood-based biomarkers, which are particularly important for developing ante mortem diagnostic methods. There are initiatives like CLARiTI that aim to enhance the ability to study the neurobiology of AD and other dementia using advanced imaging techniques such as MRI and PET, creating a standardized protocol for the analysis of imaging and blood biomarkers, facilitating data sharing among researchers and improving medical practice (For Clarity in ADRD Research Through Imaging, 2023). Research on blood biomarkers has also been limited compared to tissue-based studies. Blood biomarkers might be less specific and sensitive than those derived from brain tissues, with systemic factors influencing their levels and complicating interpretation. Increasing research on blood biomarkers through advanced proteomics and metabolomics techniques, and longitudinal studies correlating these biomarkers with Alzheimer's clinical progression, could validate their diagnostic utility. Combining blood biomarker data with clinical and omic data could improve diagnostic accuracy and disease monitoring. Integrating multi-omics results with various available databases within the same system will enable the identification of variables that may influence specific Alzheimer's phenotypes. This comprehensive strategy not only advances our understanding of the disease but also aids in the development of more targeted and effective diagnostic and therapeutic interventions.

As omics technologies continue to evolve, their integration with clinical research will be paramount in driving forward the field of neurodegenerative disease research and ultimately improving the lives of those afflicted by these debilitating conditions.
