# Supplementary material for: The ROSMAP project: aging and neurodegenerative diseases through omic sciences
Source: Front Neuroinform. 2024 Sep 16;18:1443865. doi: 10.3389/fninf.2024.1443865 (PMC11439699; doi:10.3389/fninf.2024.1443865)
Supplement: Supplementary file 1 [file Data_Sheet_1.zip › Supplementary_documents-The ROSMAP Project: Aging and Neurodegenerative Diseases through Omic Sciences/ROSMAP_review-SupplementaryMaterials.pdf]

The ROSMAP Project: Aging and Neurodegenerative Diseases through Omic Sciences

Supplementary Material

To conduct a meta-analysis of scientific contributions related to the ROSMAP project, we utilized the Entrez API from PubMed Central (PMC) to retrieve articles that incorporate the ROSMAP cohort in Alzheimer's disease research. Additionally, we extracted Medical Subject Headings (MeSH) terms—controlled, hierarchically organized vocabulary produced by the National Library of Medicine, which is used for indexing, cataloging, and searching biomedical and health-related information—from articles focused on Alzheimer's disease that include and do not include the ROSMAP cohort. A similarity matrix was then constructed to compare these MESH terms with their associated PMIDs. This methodology facilitated the development of two bipartite networks (see Figures S1 and S2). In these networks, red nodes represent MESH terms, while blue nodes denote PMIDs. The connections between PMIDs are not direct; rather, they are inferred from their shared MESH terms. Thus, the degree of a MESH term—its number of connections—reflects its prominence and the extent to which it is covered across the articles. This degree serves as a measure of centrality and connectivity, shedding light on the significance and interplay of the scientific concepts represented by the MESH terms.

There are 23 MESH terms present in the ROSMAP MESH network (Figure S1) but absent in the no-ROSMAP network (Figure S2), including *Cyclin-Dependent Kinases, Co-Repressor Proteins, ATPases Associated with Diverse Cellular Activities, Electron Transport Complex III, Arteriolosclerosis, Pharmacoepidemiology, Receptors AMPA, Schizophrenia Treatment-Resistant, Chitinases, Synaptosomal-Associated Protein 25, Receptor-Like Protein Tyrosine Phosphatases Class 2, Synaptotagmin I, Mosaicism, Sex Chromosomes, Sensory Gating, Cation Transport Proteins, Gray Matter, Plakophilins, Sweetening Agents, Taste Perception, Anxiety Disorders, Genetic Structures* and *Abdominal Pain.* Their relative absence in the no-ROSMAP network highlights a potential gap in general Alzheimer's research, where such focused investigations might be less prevalent. Conversely, these terms potentially reflect the specific research questions driven by the availability of rich phenotypic and omic data, suggesting a focus on detailed mechanistic studies within the ROSMAP cohort. This divergence underlines the importance of specific cohort studies, especially to uncover complex details of pathologies that stand out when there is representativeness of specific characteristics.

In the bipartite network depicting PMIDs and associated MESH terms from articles utilizing the ROSMAP cohort (Figure S1), the MESH node with the highest degree is *Human*, which is unsurprising given that ROSMAP exclusively comprises human subjects. Other high-degree MESH terms include *Alzheimer Disease, Genome-Wide Association Study, Brain, Female, Male, Aged, Polymorphism, Single Nucleotide, Aged 80 and over, Proteome, Transcriptome, Gene Expression Profiling, Amyloid beta-Peptides, Genetic Predisposition to Disease, Apolipoprotein E4, Quantitative Trait Loci, Epigenesis, Genetic, Middle Aged, Cognitive Dysfunction* and *Reactive Oxygen Species. Alzheimer Disease, Brain, Female, Male, Aged* are also unsurprising. Terms associated with omics sciences, such as *Proteome, Transcriptome*, and *Gene Expression Profiling* are represented, highlighting the research focus on RNA within the ROSMAP cohort. Similarly, *Genome-Wide Association Study, Polymorphism, Single Nucleotide, Genetic Predisposition to Disease, Gene Expression Profiling* and *Genetic* tell us about the prevalence of genomic research within the cohort. This indicates a strong emphasis on both genetic and transcriptomic approaches in the study of Alzheimer's disease within the ROSMAP cohort. The inclusion of terms like *Apolipoprotein E4* and *Amyloid beta-Peptides* further underscores the focus on key molecular mechanisms associated with Alzheimer's pathology. The presence of terms related to

aging (*Aged, Aged 80 and over, Middle Aged*) and cognitive decline (*Cognitive Dysfunction*) reflects the cohort's demographic and clinical relevance to Alzheimer's research. Overall, the network reveals a comprehensive approach to studying Alzheimer's disease, integrating diverse omics technologies and highlighting the multifaceted nature of research conducted within the ROSMAP framework. For further exploration of the semantic architecture of ROSMAP, the GraphML file archive is available.

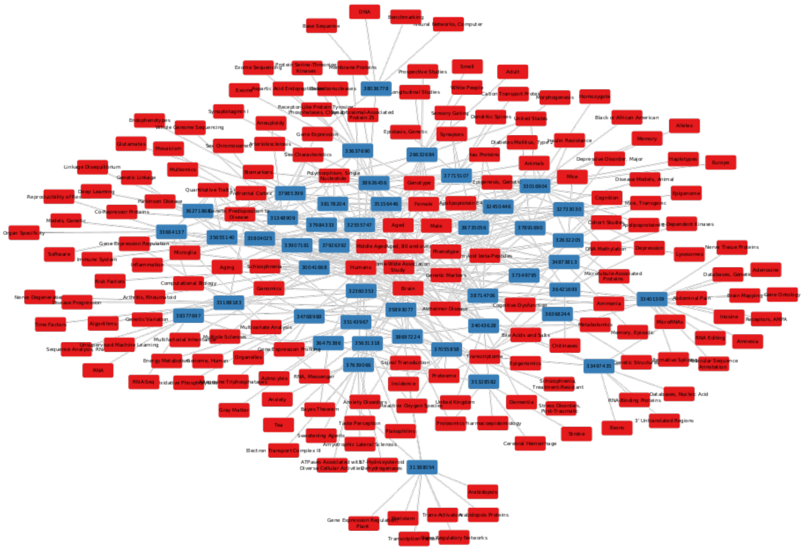

Figure S1: A bipartite network depicting PMIDs and associated MESH terms from articles utilizing the ROSMAP cohort to study Alzheimer's disease.

Table S1: MESH terms with higher degrees within the network of articles that utilize the ROSMAP database, indicating the number of connections each term has with other nodes in this network.

**MESH Term**	**Degree**
Humans	44
Alzheimer Disease	29
Genome-Wide Association Study	22
Brain	21
Female	18
Male	16
Aged	13
Polymorphism, Single Nucleotide	12
Aged, 80 and over	9
Proteome	8
Transcriptome	8
Gene Expression Profiling	7
Amyloid beta-Peptides	6
Genetic Predisposition to Disease	6
Apolipoprotein E4	5
Quantitative Trait Loci	5
Epigenesis, Genetic	4
Middle Aged	4
Cognitive Dysfunction	4
Reactive Oxygen Species	4

The no-ROSMAP network (Figure S2) is notably larger, reflecting a broader scope of Alzheimer's disease research beyond ROSMAP. High-degree MESH terms, such as *Proteomics, Humans, Proteome, Gene Expression Profiling, Animals, Transcriptome, Metabolomics, Metabolome, Mass Spectrometry,*

Alzheimer Disease, Female, Male, Biomarkers, Tandem Mass Spectrometry, Mice, Chromatography, Liquid, Computational Biology, Genomics, Signal Transduction, and *Algorithms*, highlight popular research topics. *Proteomics* and *Proteome* emerge as key terms, alongside *Humans*, underscoring the prominence of proteomics in Alzheimer's research. Other omics-related terms like *Gene Expression Profiling, Transcriptome, Metabolomics, Computational Biology*, and *Genomics* further emphasize the strong focus on multi-omics approaches. The presence of *Mice* signifies the importance of animal models in this field. The inclusion of terms like *Single-Cell Gene Expression Analysis* and *Single-Cell Analysis* reflects the growing adoption of advanced omic technologies. This underscores the evolving landscape of Alzheimer's research, where emerging techniques are increasingly contributing to our understanding of the disease. As with the first graph, the GraphML file archive is available in the supplementary materials.

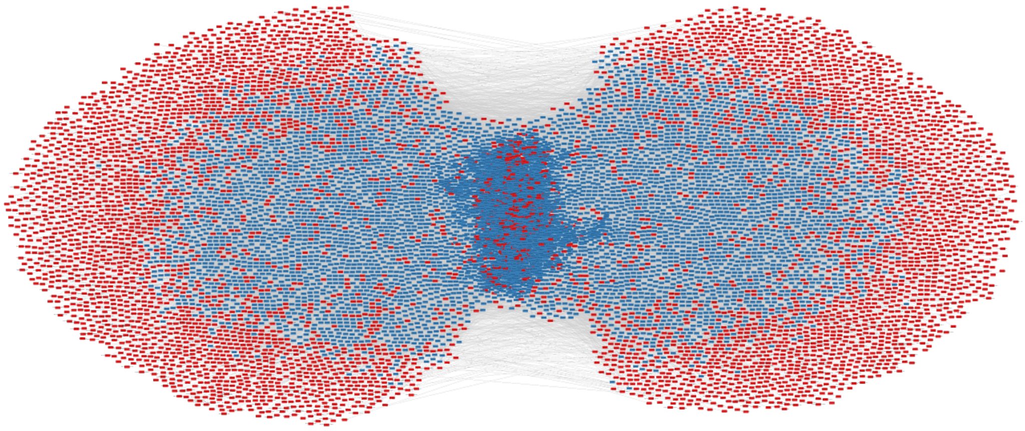

Figure S2: This bipartite network visualizes PMIDs and associated MESH terms from articles studying Alzheimer's disease without utilizing the ROSMAP cohort.

Table S2: MESH terms with higher degrees within the network of articles that do not utilize the ROSMAP database, indicating the number of connections each term has with other nodes in this network.

**MESH Term**	**Degree**
Proteomics	4675
Humans	4158
Proteome	4011
Gene Expression Profiling	3519
Animals	2646
Transcriptome	2612
Metabolomics	2267
Metabolome	1930
Mass Spectrometry	1079
Alzheimer Disease	1052
Female	961
Male	958
Biomarkers	893
Tandem Mass Spectrometry	724
Mice	722
Chromatography, Liquid	656
Computational Biology	654
Genomics	623
Signal Transduction	532
Algorithms	461

The shared terms between the two lists of higher-degree nodes — *Humans, Proteome, Alzheimer Disease, Female* and *Male*—reflect fundamental concepts central to both sets of articles. These terms represent core aspects of research in neurodegenerative diseases and human biology, suggesting that AD research has a common focus on understanding the human proteome and Alzheimer's disease, focusing on both male and female subjects, irrespective of the specific cohort used. In contrast, terms present in the higher-degree node term list of articles that did not utilize the ROSMAP cohort but absent from the higher-degree node list of articles using ROSMAP include *Animals, Metabolomics, Metabolome, Mass Spectrometry, Biomarkers, Tandem Mass Spectrometry, Mice, Chromatography Liquid, Computational Biology, Signal Transduction*, and *Algorithms*. Articles that do not use the ROSMAP cohort might focus on broader or different aspects of biological and medical research, including metabolomic studies or investigations involving animal models. Terms like *Animals* and *Mice* are often associated with experimental techniques that are be more prevalent in studies using non-human models or different types of experimental designs.

While shared terms reflect common areas of interest, the unique terms present in each network underscore the specific aspects of research and experimental focus associated with each set of studies. Understanding these differences can provide insights into how the choice of research cohort influences the scope and depth of scientific investigation in the field of neurodegenerative diseases.
